# Supplementary material for: Matching Mechanics and Energetics of Muscle Contraction Suggests Unconventional Chemomechanical Coupling during the Actin–Myosin Interaction
Source: Int J Mol Sci. 2023 Aug 1;24(15):12324. doi: 10.3390/ijms241512324 (PMC10418673; doi:10.3390/ijms241512324)
Supplement: Supplementary file 1 [file ijms-24-12324-s001.zip › ijms-2502819-supplementary.pdf]

Supplementary Material

Thermodynamic compatibility for an attached myosin motor to slip to the next 5.5 nm actin monomer during shortening

The mechanical-kinetic model of the actin-myosin ATPase cycle during contraction of Ca^{2+}-activated skinned fibres from rabbit psoas reported in [35] contains the demonstration of the thermodynamic compatibility of the possibility of an attached motor to slip to the next actin monomer 5.5 nm away from the centre of the sarcomere. The model was developed to explain the transient and steady state responses to different loads and their modulation by Pi concentration. In that model the working stroke of the attached motor can occur in either the AM.ADP.P_i or the AM.ADP state and implies three subsequent structural transitions or force-generating steps among four structural states (identified by the thickness of the line in Figure S1), controlled by strain-dependent rate constants.

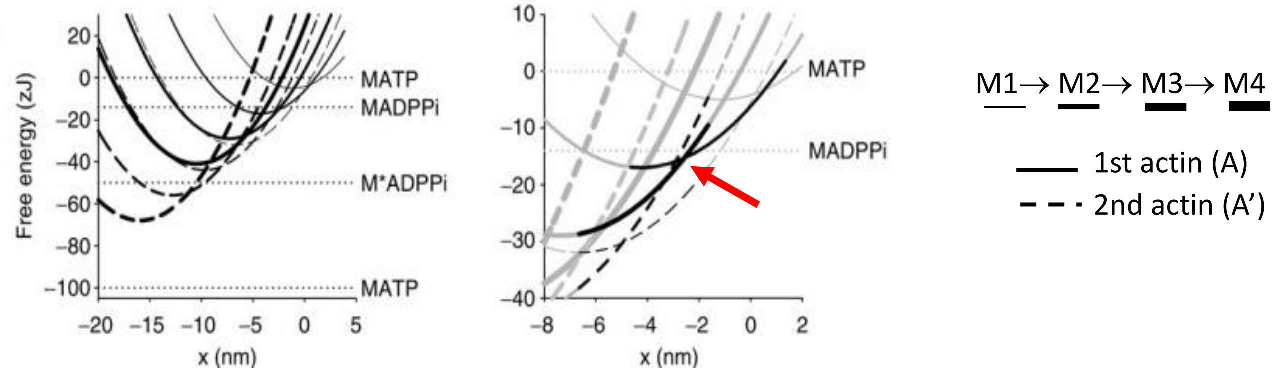

Figure S1. Thermodynamic compatibility for an attached myosin motor to slip to the next 5.5 nm actin monomer during shortening. Left panel: free energy diagrams of the four structural states of the myosin motor in the AM.ADP.Pi state, attached to either the original actin monomer (A, continuous lines) or the next actin monomer 5.5 nm farther from the centre of the sarcomere (A', dashed lines). The thickness of the line identifies the progression of the state transitions, from M1, thinnest line, to M4, thickest line. The slip from A to A' corresponds to a leftward 5.5 nm shift and a downward 27 zJ shift of the minimum of the free energy parabola of the motor in each state; *x* is zero in correspondence of the free energy minimum of a myosin motor attached to A in the M1 state. Horizontal lines show the free energy of the detached states. Right panel: shown in grey are the same diagrams as in the left panel, but on an expanded scale to show the energy profiles in the x range concerning the slipping transition. Shown in black are the free energy profiles of the states populated during steady shortening at a velocity (<1000 nm s^{-1}) that maximises the slipping process. In correspondence of the red arrow, the A-attached M_2 motor (thinner continuous line) dwells between the transition to the M_3 state (thicker continuous line) or the slipping to A' (dashed line with the same thickness); the increase in strain following the slipping favours the backward state transition to M_1 (thinnest dashed line). Adapted from Figure 4 in [35], reproduced with permission.

In that paper (*i*) the stiffness of the myosin motor sets the constraint for the size of the structural transition (3.1 nm) and thus the number of transitions (3) to account for both the transients following

length or force steps and the overall size of the working stroke (10 nm); (*ii*) both biochemical events in the attached motor, release of P_i and release of ADP, can occur in any of the four states, the rate constants of P_i release and ADP release increasing with the progression of the motor through the working stroke from M_1 to M_4.
